# Biosensor nanoarchitectonics of Cu–Fe-nanoparticles/Zeolite-A/Graphene nanocomposite for enhanced electrooxidation and dopamine detection

**DOI:** 10.1016/j.heliyon.2023.e19741

**Published:** 2023-09-01

**Authors:** Navashree Nagarajan, Parthasarathy Panchatcharam

**Affiliations:** a SERB, Department of Electronics and Communication Engineering, CMR Institute of Technology, Bengaluru, 560037, India; b Department of Electronics and Communication Engineering, CMR Institute of Technology, Bengaluru, 560037, India

**Keywords:** Bimetallic nanoparticles, Zeolite, Nano-hybrid materials, Dopamine detection, Electrochemical sensing

## Abstract

Cu–Fe NPs/ZEA/Gr electrochemical biosensor is developed by sol-gel spin coating technique, where copper-iron nanoparticles (Cu–Fe NPs) is synthesized using a chemical reduction method and modified with Zeolite & Graphene to develop a hybrid nanocomposite - Cu–Fe NPs/ZEA/Gr. The synthesized nanocomposite is then mixed with poly (vinyl alcohol) as a binding agent and coated on to the glass substrate to produce thin film electrode. Then the electrode was analyzed for structural and morphological studies using XRD, SEM, TEM, UV-VIS, absorption, and emission spectra. The presence of Cu–Fe NPs, ZEA, and Gr in the nanocomposite is confirmed by the XRD diffraction peaks, while SEM investigation revealed that the hybrid composite has a particle size of around 7.25 nm with a body-centred cubic structure. The TEM images show that bimetallic nanoparticles were incorporated into the ZEA shell, which was surrounded by a layer of transparent graphene. Furthermore, the nanocomposite exhibited a distinct absorption peak at 395 nm, as evidenced by UV-VIS, absorption, and emission spectra. The electrochemical tests demonstrated that the Cu–Fe NPs/ZEA/Gr nanocomposite electrode showed an excellent electrocatalytic and selective properties towards the electrooxidation of dopamine to dopamine-o-quinone. The detection limit of the Cu–Fe NPs/ZEA/Gr nanocomposite thin film was found to be 0.058 μM, with a sensitivity of 1.97 μAμM^−1^cm^−2^. The enhanced catalytic performance of the Cu–Fe NPs/ZEA/Gr electrode is attributed to the unique nanostructured materials coating on the glass substrate. The findings suggest that nano-hybrid materials can be a viable option for developing electrochemical biosensors to monitor dopamine levels in biological fluids. This indicates that the concept of nanoarchitectonics utilized to produce dopamine sensors may lead to new diagnostic and therapeutic approaches for neurological disorders associated with dopamine dysregulation.

## Highlights

• A novel Cu–Fe NPs/ZEA/Gr modified electrode for dopamine estimation.
• Methods for the design and development of sensitive nanoparticles based sensors.
• Significant enhancement of current vs. voltage peak was observed.
• Provides challenges and opportunities for future nanoparticle based graphene sensors.

## Graphical abstract

**Figure:**
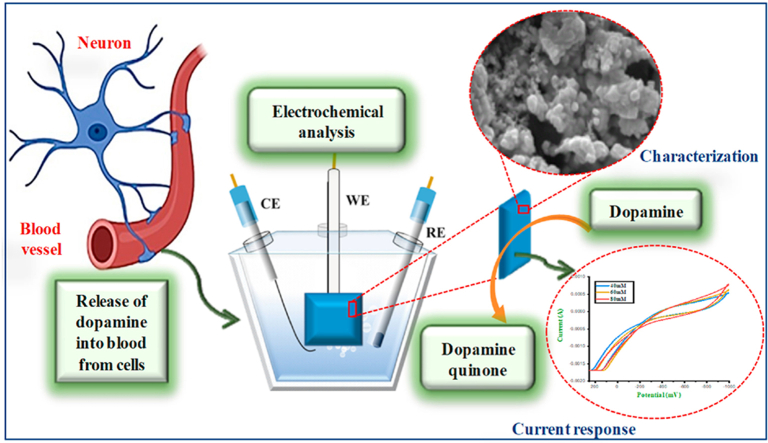

## 1 Introduction

**Fig. 1:**
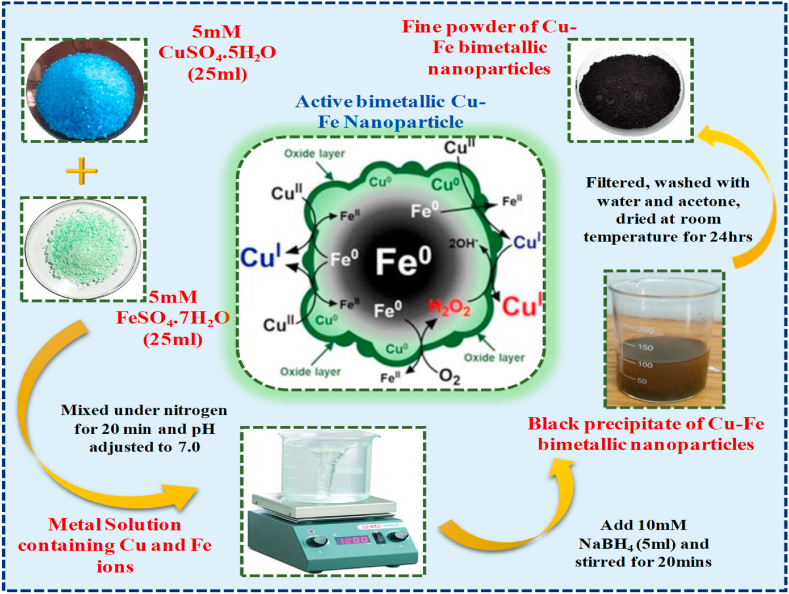
Diagrammatic representation for Cu–Fe bimetallic nanoparticles synthesis.

The human metabolic process is significantly influenced by numerous biomolecules that serve as signaling molecules and catalysts for various processes and functions. Neuroactive molecules and neurotransmitters like dopamine play a key role as chemical messengers. Dopamine is mainly located in major brain and muscle sites and acts as a natural neurotransmitter for various motor functions. It is part of the catecholamine family, derived from phenylalanine or tyrosine, and has the chemical formula of 3,4-dihydroxy phenylethylamine. It regulates the human physiological and psychological responses such as movement, mood, behavior, learning, and memory. The dopamine level can be used to diagnose various pathological conditions, including cancer and neurological disorders. A decreased dopamine level leads to Parkinson's disease and schizophrenia [1]. These dopamine levels are also increased during anxiety and depression. Thus, the unbalanced level of dopamine in the body should be measured which is required for understanding its biological mechanism and functions. Previously there were numerous methods such as high-performance liquid chromatography-mass spectrometry (HPLC-MS) and ultrahigh performance liquid chromatography-electrospray ionization-tandem mass spectrometric method (UHPLC/ESI-Q-TOF-MS) for the detection of dopamine levels. However, researchers considered that these procedures are time-consuming and difficult for continuous monitoring of dopamine. Nowadays, more attention has been paid to electrochemical techniques for the rapid detection of dopamine due to its advantageous simple operation, low cost, high sensitivity, fast response, good stability, enhanced reproducibility, and minimum power requirement [2,3] (see Fig. 1).

Numerous materials have been used for the development of a sensor for the detection of dopamine. In particular, graphene (Gr) is a brand new one-atom-thick two-dimensional graphic carbon structure that has emerged as a rising star in the field of materials research. It is popular among other materials because of its distinctive structural and electrical properties in comparison with the other carbon-based materials and also provides the best microenvironment for the immobilization of various biomolecules and electron transmission. For the electrochemical analysis of dopamine, a modified electrode based on graphene has demonstrated good sensitivity and stability. On the other hand, due to unique cation exchange and electrocatalytic properties, zeolite-modified electrodes have recently gained attention for electrochemical applications. Significant effort has been put into the development of zeolite-modified electrodes (ZME) based biosensors. ZMEs can be loaded with metal ions or metal nanoparticles due to their advantageous three dimensional lattices possessing a variety of attachment sites [4]. Among all these materials nowadays nanoalloys have been used in various fields for their great scientific and technological importance. Especially bimetallic nanoparticles have the potential to control the morphology of their nanostructure [5,6]. Thus, the sensitivity of the biosensor can be improved by the application of Cu–Fe bimetallic nanoparticles for the detection of dopamine. In numerous metals, copper is a mild hydrogenation catalyst with low cost when compared to other noble metals [7]. It is incorporated with iron to produce the Cu–Fe bimetallic nanoparticles by the facile one-pot chemical synthesis. However, a study also revealed that the affinity of the Cu–Fe binding is higher when compared with the other precious metals and possesses high reactivity [8,9]. Considering the unique properties of zeolite and graphene (Gr), we presented the Cu–Fe bimetallic nanoparticles doped ZEA (Zeolite A) and Gr (Cu–Fe NP/ZEA/Gr) modified electrode for the electrochemical determination of dopamine neurotransmitter.

## 2 Experimental

### 2.1 Reagents and materials

Copper sulphate (CuSO_4_.5H_2_O), Ferrous sulphate (FeSO_4_.7H_2_O), Sodium borohydride (NaBH_4_), Sodium aluminate (Na_2_O·Al_2_O_3_), Sodium metasilicate (Na2SiO3·5H2O), Sodium hydroxide (NaOH), Graphene, Poly-vinyl alcohol (PVA) were purchased from Kesari Scientific Chemicals, Chennai. All the chemicals were purchased as analytical grade and used without any further purification process. A phosphate buffer solution was prepared by using Sodium chloride (NaCl), Potassium chloride (KCl), Sodium phosphate dibasic (Na_2_HPO_4_), and Potassium phosphate monobasic (KH_2_PO_4_) with 7.4 pH. The stock solution of dopamine was prepared by using Dopamine hydrochloride on daily basis. Double distilled water was used throughout the experiment.

### 2.2 Apparatus and measurements

X-ray diffraction (XRD) measurements were performed on X’ Pert PRO X-ray powder diffractometer (Netherlands) with Cu K radiation (0.154060 nm) and recorded from 10° to 80° at a speed of 2° per minute. The surface morphology was studied using the Field Emission Scanning Electron Microscope (FESEM) (FEI Quanta 200) and Field Emission Transmission Electron Microscope (FETEM). The transmittance spectra in the wavelength range of 200–800 nm were recorded using a JASCO UV-VIS-NIR (V670) spectrometer. The data of Cyclic voltammetry (CV), and differential pulse voltammetry (DPV) was measured using ZENSOR electrochemical workstation (USA) with a conventional three-electrode system composed of a platinum electrode as counter electrode, Ag/AgCl electrode as reference electrode, and a modified bare electrode (diameter = 3 mm) as working electrode.

### 2.3 Preparation of Cu–Fe nanoparticles

Copper-iron bimetallic nanoparticles (NPs) were prepared by mixing 5 mM CuSO_4_.5H_2_O and FeSO_4_.7H_2_O solutions. 100 ml of each metallic solution were mixed under nitrogen for 20 min and pH was adjusted to 7.0. Add 20 ml of 10 mM NaBH_4_ dropwise to the metal solution and stirred for 10 min, a black precipitate was obtained and filtered. Then it is washed with deionized water and finally by acetone to remove the water completely from the precipitate. It is dried at room temperature for 24 h to get powdered Cu–Fe bimetallic nanoparticles [5,10].

### 2.4 Process of zeolite synthesis

**Fig. 2:**
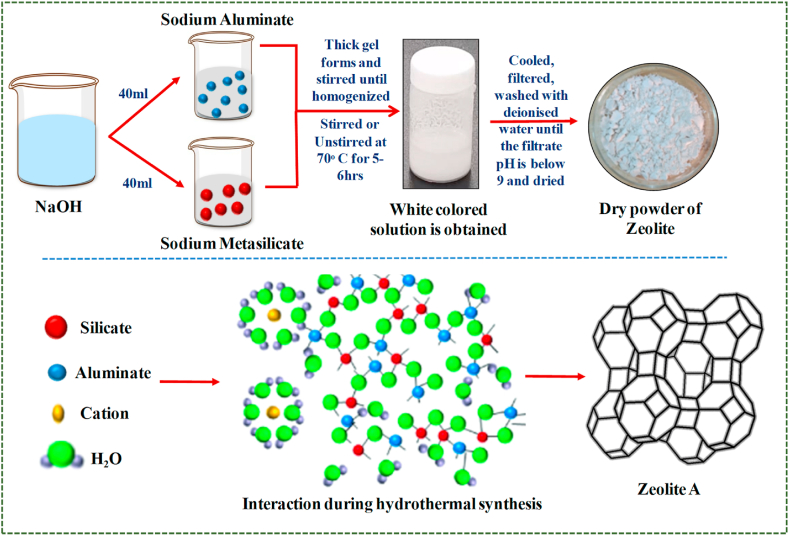
Process flow for Zeolite A synthesis.

Zeolite is a pure white crystalline powder produced by the traditional batch culture method. 0.723 g of NaOH was mixed with 80 ml of deionized water and the solution was divided into two equal halves. To one half of the solution, add 8.258 g of sodium aluminate and mix gently in the capped bottle until the solution is clear. Add 15.48 g of sodium metasilicate to the second half of the NaOH solution and mix gently until clear. Pour silicate solution into aluminate solution quickly, a thick gel forms, then cap tightly and mix until homogenized. Then the mixture was kept in 70ᵒC for 5–6 h to obtain a pure white solution. It is cooled, filtered, washed with deionized water until the filtrate pH is below 9 and dried at room temperature for 48 h to get a pure dry crystalline zeolite powder [11] (see Fig. 2).

### 2.5 Cu–Fe NP/zeolite modified graphene

250 mg of Cu–Fe NPs were added to 20 ml of water and stirred for 15 min to produce the homogenous mixture. To this mixture add 1.0 g of Zeolite, then stirred continuously for 12 h s at room temperature. The solution was filtered and dried to get a dry Cu–Fe NPs/ZEA. To 50 mg of Cu–Fe NP/ZEA add 20 ml of water and stirred for 10 min, to this 10 mg of graphene nanopowder was dispersed with continuous stirring for 10 min. To the mixture add 5 ml of 30 mM NaBH_4_ and stirred at 65ᵒ C for 5–6 h which produces a black precipitate of Cu–Fe NP/ZEA/Gr. It is cooled, filtered and, dried at room temperature for 24 h to produce the dry powder [4,12].

### 2.6 Fabrication of Cu–Fe NP/Zeolite modified graphene thin film electrode

To prepare a Cu–Fe NP/ZEA/Gr sol, 10 mg of dry Cu–Fe NP/ZEA/Gr was added to the 10 ml of PVA solution (5% PVA solution) and stirred for 3–4 h. For the fabrication of the electrode, the precleaned bare glass was treated with deionized water and acetone to remove the impurities on the surface and placed in a spin coater. The prepared sol was coated using the spin coating method on the clean glass slide and dried at 250ᵒC for a few minutes in a hot air oven to produce a uniform modified thin film over the bare glass. The produced Cu–Fe NP/ZEA/Gr thin film was used as a working electrode for the electrochemical analysis of dopamine.

**Fig. 3:**
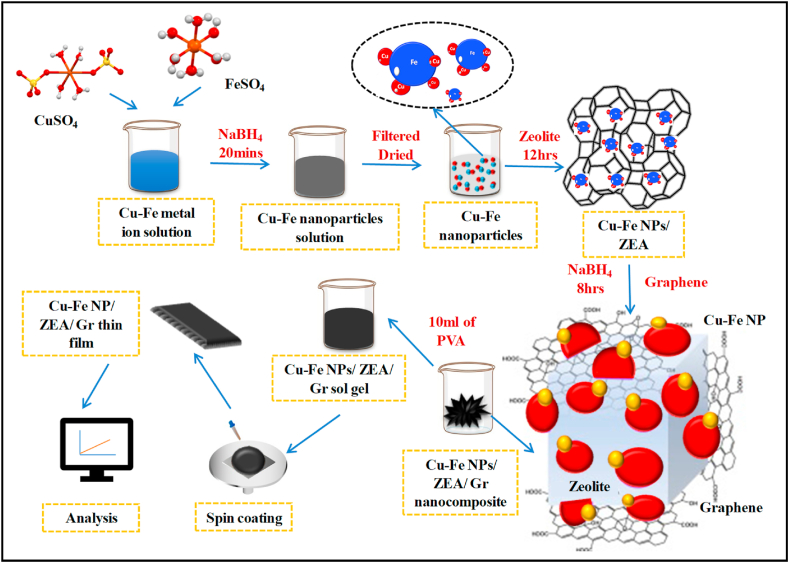
Overall process for the development of Cu–Fe NPs/ZEA/Gr thin film.

For the first time, a Cu–Fe NP/ZEA/Gr thin film with unique characteristics was successfully synthesized and used to develop an electrochemical sensor for the electrochemical detection of DA. The electrode fabrication was fascinating and yet simple method to follow. In terms of sensitivity, detection thresholds, linear range, and stability, CV presents and discusses the electrochemical performance of the produced sensor. The study might provide a brand-new manufacturing platform for zeolite- and nanoparticle-based electrochemical sensors (see Fig. 3).

## 3 Result and discussion

### 3.1 Material characterization (XRD, SEM, TEM, UV, adsorption and emission spectra)

**Fig. 4:**
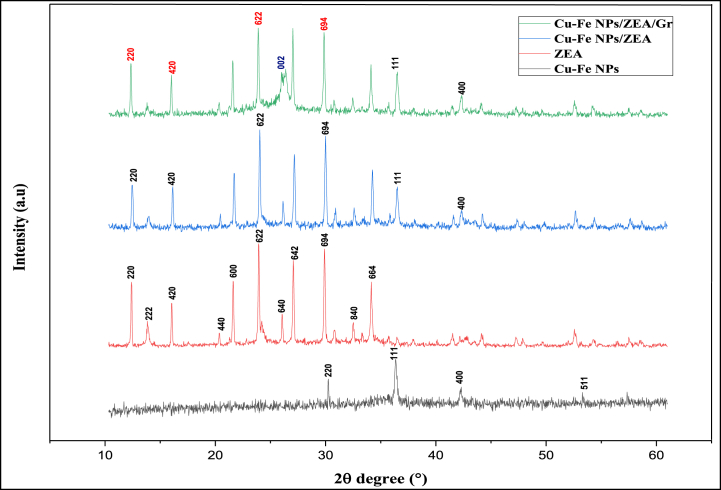
XRD pattern for Cu–Fe NPs, ZEA, Cu–Fe NPs/ZEA, Cu–Fe NPs/ZEA/Gr nanocomposite.

Characterization of Cu–Fe NPs, Cu–Fe NPs/ZEA, and Cu–Fe NPs/ZEA/Gr was examined by using XRD analysis which was shown in Fig. 4. After the chemical reduction by NaBH_4,_ diffraction peaks were observed at 30.2ᵒ (220), 43.3ᵒ (400) and 57.3ᵒ (511) corresponds to ferric oxide whereas the sharp peak at 36.4ᵒ (111) corresponds to copper oxide which confirms the formation of Cu–Fe bimetallic nanoparticles. This is well agreed with the previous work of various researchers [4,6,13]. The highest peak at 36.4ᵒ represents the highest percentage of copper oxide than ferric oxide. Chemically synthesized Zeolite A (ZEA) has been characterized using XRD in which the diffraction peaks at 10.4ᵒ (220), 12.8ᵒ (222), 16.5ᵒ (420), 21.6ᵒ (440), 24ᵒ (600), 26.2ᵒ (622), 27ᵒ (640), 30ᵒ (642), 31ᵒ (694), 32.5ᵒ (840) and 34.3ᵒ (664) confirms the formation of pure ZEA composed of AlO_2,_ SiO_2_ strongly bonded with sodium ions. This is in agreement with the work of previous studies [14,15]. When Cu–Fe bimetallic nanoparticles are combined with ZEA, it shows similar diffraction peaks corresponding to the compounds. The diffraction peaks of both Cu–Fe NPs and ZEA can be seen in the result. However, the newly synthesized Cu–Fe NPs/ZEA modified Graphene (Gr) shows the diffraction peaks at 10.4ᵒ (220), 12.8ᵒ (222), 16.5ᵒ (420), 21.6ᵒ (440), 24ᵒ (600), 26.2ᵒ (622), 27ᵒ (640), 30ᵒ (642), 31ᵒ (694), 32.5ᵒ (840), 34.3ᵒ (664) 36.4ᵒ (111) and 43.3ᵒ (400) which corresponds to the Cu–Fe NPs/ZEA. In addition to these diffraction peaks a broad peak at 26.6ᵒ (002) represents the presence of graphene. It confirms the formation of Cu–Fe NPs/ZEA/Gr nanocomposite. It was observed from Fig. 4 the peak positions of ZEA had no significant changes after the addition of Cu–Fe NPs and graphene which inferred that these compounds are well dispersed in the zeolite framework. In addition, the XRD spectrums are in agreement with a body-centred cubic (bcc) structure which matches the JCPDS Card No. JCPDS-039-0222.

**Fig. 5:**
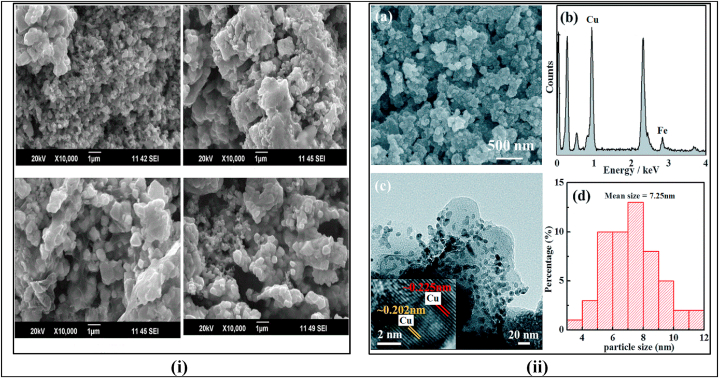
(i) and 5 (ii): SEM images of Cu–Fe NPs/ZEA/Gr nanocomposite thin film.

The SEM analysis of the newly developed Cu–Fe NPs/ZEA/Gr thin film was conducted to examine the morphological properties of the nanocomposite, such as its size and structure. The results, shown in Fig. 5(i) and (ii), indicate that the Cu–Fe bimetallic nanoparticles are well dispersed throughout the ZEA network and present in the form of cubic crystals. The ZEA particulates have a porous structure, and the Cu–Fe bimetallic nanoparticles are loaded onto them without altering the morphology of either component. Additionally, it was found that the Cu–Fe NPs/ZEA nanocomposite aggregates well with graphene as polycrystalline structures with distinct edges. The particle size of the newly developed Cu–Fe NPs/ZEA/Gr nanocomposite was determined to be 7.25 nm, as shown in Fig. 5(ii).

**Fig. 6:**
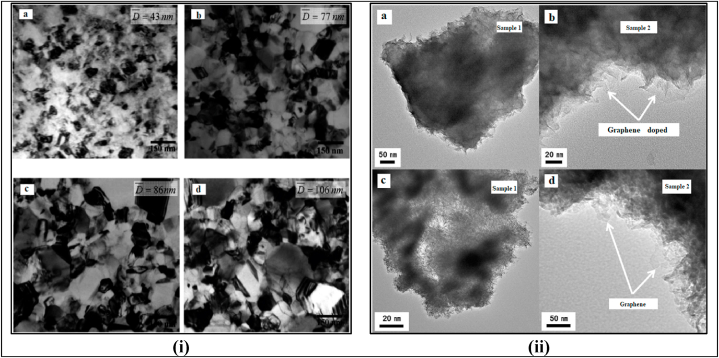
(i) and 6 (ii): TEM images of Cu–Fe NPs/ZEA/Gr nanocomposite thin film.

The TEM images of the Cu–Fe NPs/ZEA/Gr thin film are shown in Fig. 6(i) and (ii). At low magnification, Fig. 6(i) shows the uniform and well-defined nanosized cubic nanoparticles of Cu–Fe that are integrated with the ZEA and graphene network. The higher magnification image reveals the core structure of the nanoparticles within the ZEA shell, with the darker contrast areas indicating the presence of Cu–Fe bimetallic nanoparticles within the pores of the ZEA. Fig. 6(ii) compares the Cu–Fe NPs/ZEA thin film (sample-1) with the Cu–Fe NPs/ZEA/Gr thin film (sample-2). It is observed that sample-2 has a thin transparent layer which is absent in sample-1, and represents the graphene that has been successfully incorporated into the nanocomposite. These results are consistent with the findings by the previous researchers [4,16] and from the SEM and XRD analysis of this study.

**Fig. 7:**
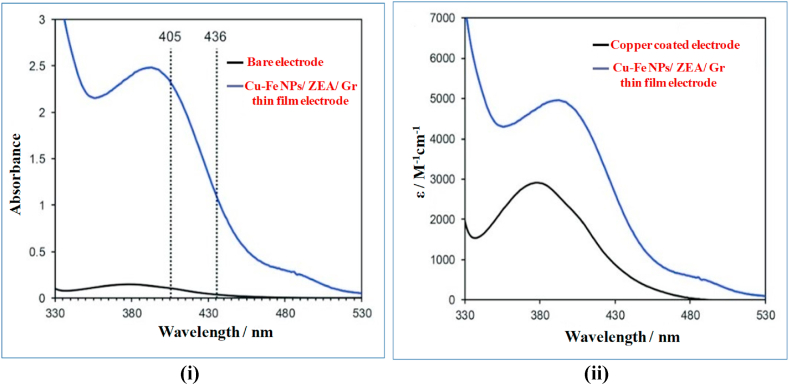
(i) &7 (ii): UV-VIS pattern of Cu–Fe NPs/ZEA/Gr nanocomposite thin film.

Each material has different range of absorption for UV- VIS spectrum. Fig. 7(i) &7 (ii) shows the UV-VIS spectrum of the developed Cu–Fe NPs/ZEA/Gr thin film electrode. In Fig. 7(i), the Cu–Fe NPs/ZEA/Gr thin film electrode was compared with the bare electrode. The results show that there is no significant absorbance peak when the incident light is passed through the bare electrode whereas when the same amount of incident light is passed through the newly developed electrode it ultimately produces an absorbance peak at 395 nm which is unique for this nanocomposite. This absorbance peak is conjugated with the different materials (Cu–Fe nanoparticles, Zeolite A, and Graphene) present in the nanocomposite. After the maximum absorbance of light by the chemical substance, there is a gradual decrease in the peaks from 405 nm to 436 nm. The maximum absorbance is due to the surface plasma resonance demonstrating the developed nanocomposite is highly reactive and stable with no significant variance in the size and shape which is consistent with the XRD, SEM, and TEM characterization results. Meanwhile in Fig. 7(ii), the UV-VIS for copper-coated electrode and Cu–Fe NPs/ZEA/Gr thin film electrode were compared. It shows the absorbance intensity is increased when compared with the copper coated electrode which suggests that the newly developed electrode contains more amount of Cu^2+^ ions and Fe^2+^ ions that can be potentially used for the electrochemical determination of biomolecules.

**Fig. 8:**
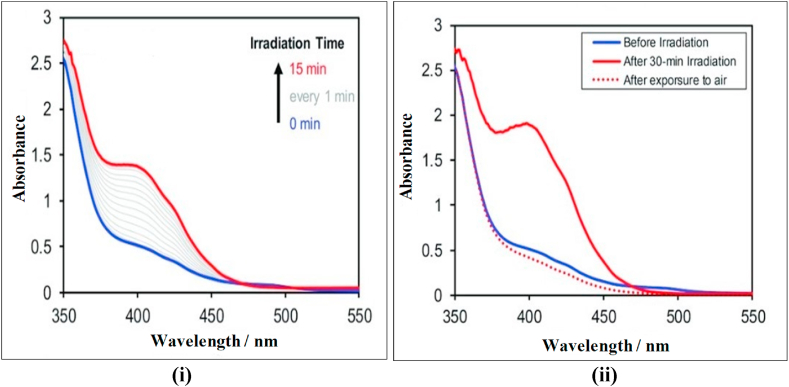
(i), 8 (ii): Absorption spectra of Cu–Fe NPs/ZEA/Gr nanocomposite thin film.

Fig. 8(i) and (ii) represents the absorption spectra of the newly developed Cu–Fe NPs/ZEA/Gr thin film electrode. From Fig. 8(i), it was observed that when a light source is passed through the electrode, the atoms present in the ground state absorbs some amount of energy to attain the exited state and resulted as absorption peak at 405 nm. At 0 min i.e., before the incidence of light on the film, the peak was not observed, but when the radiation falls on, the increase in the absorption spectra can be clearly seen for every 1 min until it reaches the maximum at 15 min.

**Fig. 9:**
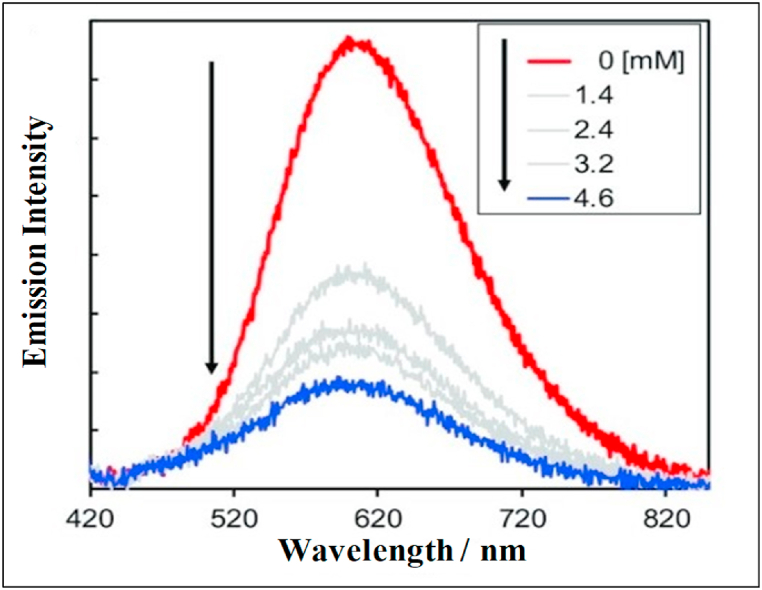
Emission spectra of Cu–Fe NPs/ZEA/Gr nanocomposite thin film.

Fig. 8(ii) shows the absorption spectra of the electrode when it is exposed to air, before and after irradiation which is consistent with Fig. 8 (i). Similarly, emission spectra of the atoms present in the electrode successfully emit the radiation by the electrons in the exited state which was shown in Fig. 9. Thus, the atoms in the thin film have a great absorption and emission capability.

### 3.2 Possible working mechanism of developed thin film

Sodium borohydride is used as a common reducing agent to form metal nanoparticles from the metal ions. The following stoichiometric equation can be used to represent the reduction of Cu(II) and Fe(II) by sodium borohydride.

(1) 4Cu^+2^ + 4Fe^+2^ + 3BH^−^_4_ + 12H_2_O → 4Cu^0^ Fe^0^ + 3B(OH)^−^_4_ + 24H^+^

Cu ions and Fe ions are reduced with NaBH_4_ to form bimetallic Cu–Fe nanoparticles. The interfacial energy nanocrystalline alloyed phases, which occur as a result of lattice deformation, and dislocations increase the free energy in the system and can drive the alloying process. Surface energy can be the driving force in alloys formed through chemical reduction.

Zeolite synthesis is a simple process in which sodium aluminate and sodium metasilicate reacts in the presence of sodium hydroxide. The mechanism of reaction is stated in the equation below.

(2) nNaO_2_.Al_2_O_3_.3H_2_O + nNa_2_SiO_3_.5H_2_O + nNaOH→ Na_n_[(AlO_2_)_n_(SiO_2_)].nH_2_O

The Cu–Fe bimetallic nanoparticles are doped onto the zeolite A as Cu–Fe/ZEA. In which the Cu–Fe nanoparticles are scattered on the surface of the zeolite without changing the lattice surface which is expressed in the equation below.

(3) nCu-Fe + Zeolite A → Zeolite A-(Cu–Fe)_n_

Zeolite A doped with Cu–Fe nanoparticles is further modified with graphene to increase the conductivity of the prepared electrode. The graphene is combined with the Cu–Fe/ZEA using a simple reduction process in which sodium borohydride acts as a reducing agent. The following equation explains the reaction mechanism.

(4) Zeolite A-(Cu–Fe)_n_ + Gr + 3BH^−^_4_ → Zeolite A-(Cu–Fe)_n_ Gr + 3B(OH)^−^_4_ + 24H^+^

**Fig. 10:**
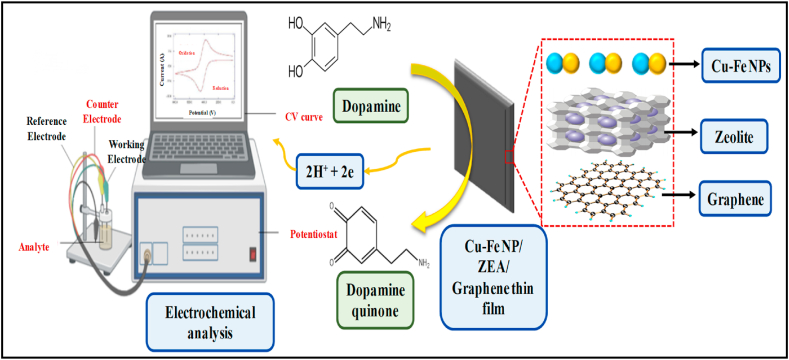
Working principle of the developed of Cu–Fe NPs/ZEA/Gr nanocomposite thin film.

The produced Zeolite A-(Cu–Fe)_n_ Gr complex is coated on the surface of the bare glass. They are covalently attached to the bare glass in the body centred cubic (bcc) pattern forming Cu–Fe NP/ZEA/Gr thin film. This newly developed electrode reacts with the hydroxyl group of the dopamine releasing 2H^+^ ions and 2e^−^ on the surface producing dopamine quinone. Each dopamine molecules thus release 2H^+^ + 2e^−^ ions when reacts with the electrode surface and produces current when the potential is applied. The schematic representation of the above-mentioned process is illustrated in Fig. 10. Likewise, when the concentration of the dopamine is increased the corresponding current value is increased which makes it possible for the quantitative determination.

### 3.3 Electrochemical analysis

**Fig: 11:**
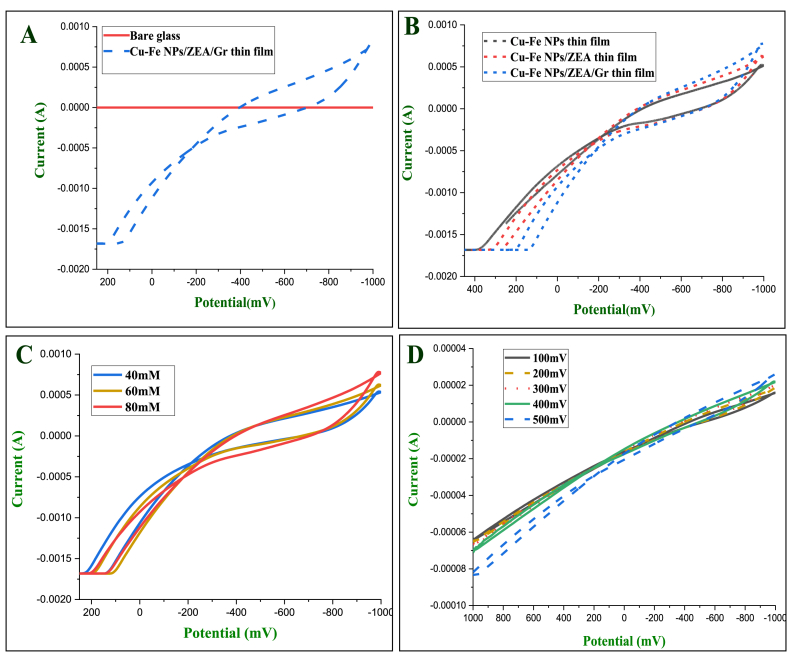
Cyclic voltammograms of dopamine with newly developed Cu–Fe NPs/ZEA/Gr thin film; A: CV of bare glass vs. Cu–Fe NPs/ZEA/Gr thin film; B: CV with different electrodes; C: CV with different concentration; D: CV with different scan rates at 20 μM.

The electrochemical behavior of the Cu–Fe NPs/ZEA/Gr thin film was analyzed using cyclic voltammetry (CV) measurements in 10 mM phosphate buffered saline (PBS) solution with a pH of 7.4 and a three-electrode system. Fig. 11 displays the CV results for dopamine at different electrode configurations, concentrations, and scan rates. To evaluate the conductivity of the newly developed electrode, it was compared to a bare glass electrode. The bare glass electrode showed no current response, while the newly created Cu–Fe NPs/ZEA/Gr thin film electrode demonstrated a significant reduction current response, as shown in Fig. 11(A). The cathodic peak observed between −1 V and 0.2 V confirmed the electrocatalytic behavior of the thin film for dopamine electrooxidation. The oxidation process started from −0.9 V outside the cathode, as shown in Fig. 10. The CV results indicate that the sensors exhibit a significant reaction to dopamine in the PBS.

#### 3.3.1 Different working electrodes

Fig. 11 (B) displays the CV results for a solution containing 80 mM dopamine (DA) at the Cu–Fe NPs, Cu–Fe NPs/ZEA, and Cu–Fe NPs/ZEA/Gr thin film electrodes. The oxidation peaks for each electrode are shown in the figure. The Cu–Fe NPs/ZEA/Gr thin film showed a larger peak current compared to the other two electrodes. The reactivity of Cu–Fe NPs with DA produced a redox curve due to its immediate electrooxidation at the electrode surface. Similarly, the addition of ZEA with Cu–Fe NPs increased the catalytic current and electrical conductivity due to its increased surface area. Whereas, larger electrocatalytic activity in the Cu–Fe NPs/ZEA/Gr thin film is due to the two-dimensional structure of ZEA and graphene. The planar hexagonal carbon structure of graphene and cuboid-shaped Cu–Fe nanoparticles allow for efficient electron transfer with the phenyl structure of dopamine. Furthermore, the increased surface area of ZEA increases the porosity and facilitates electron transfer within the electrode surface, making the Cu–Fe NPs/ZEA/Gr electrode highly efficient for the electrochemical detection of dopamine.

#### 3.3.2 Different concentration

The current response of the Cu–Fe NPs/ZEA/Gr thin film electrode for various dopamine (DA) concentrations was analyzed using CV analysis. The relationship between different concentrations of DA and oxidation current in 10 mM PBS (pH 7.4) is shown in Fig. 11 (C). The oxidation peak current was linearly proportional to the concentration of DA. This result demonstrates that the catalytic reduction current increases with increasing DA concentrations (40–80 mM), making the sensor suitable for quantitative analysis.

#### 3.3.3 Different scan rate

**Fig. 12:**
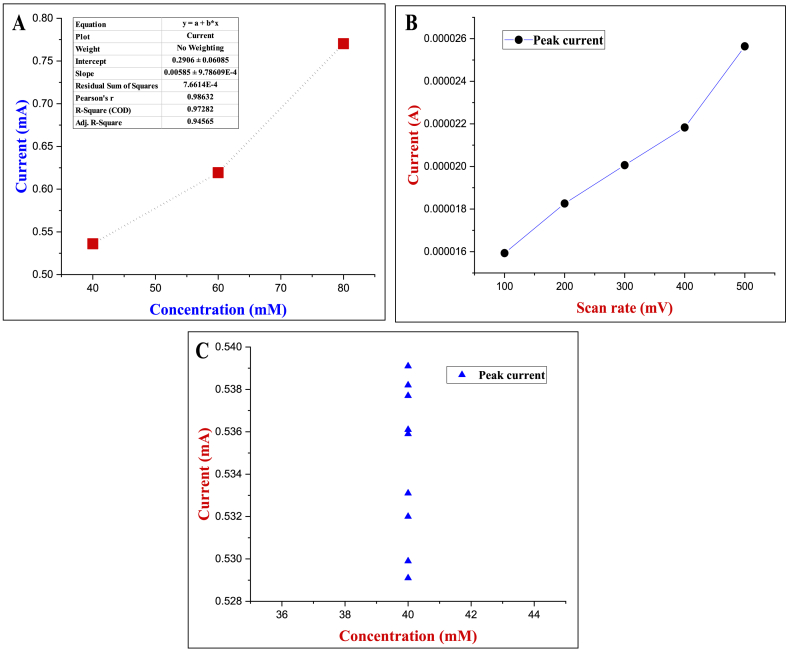
Peak current density VS dopamine concentration; A. Peak current at different concentration; B. Peak current at different scan rate; C. Peak current at 40 mM concentration.

The effect of scan rate on the electrochemical performance of the sensor was investigated by conducting CV experiments. Fig. 11 (D) shows the CVs of the Cu–Fe NPs/ZEA/Gr thin film electrode at different scan rates (100–500 mV/s) in 10 mM PBS (pH 7.4) with 20 mM concentrations of dopamine (DA). It was found that the current response increases linearly with increasing scan rate, indicating the electrooxidation of DA. Fig. 12 (B) shows the relationship between different scan rates and peak current. The correlation observed between the reduction current and the square root of the scan rate conforms to the Randles-Sevcik equation, which characterizes the performance of electrochemical systems operating in conditions that are controlled by diffusion.

#### 3.3.4 Sensitivity, slope, limit of detection (LOD) and limit of quantification (LOQ) analysis

The analytical efficiency of a Cu–Fe NPs/ZEA/Gr thin film was studied. The amperometric response for a known amount of dopamine in a stirred 10 mM PBS (pH 7.4) solution was tested at the scan rate of 500 mV/s after successive additions of a standard dopamine solution. This procedure yielded a calibration curve with a linear range of 40 mM to 80 mM which is represented in Fig. 12 (A). The calibration curves for measured amperograms are shown in Fig. 11(C). The proposed sensor has a relative sensitivity of about 1.97 μAμM^−1^cm^−2^ whereas the LOD was found to be 0.058 μM, which was calculated by using the 3S/k approach [17] and LOQ of the biosensor was also calculated which was found to be 1.78 μM. Table .1 shows a brief literature summary of electrochemical sensing performance of various developed electrodes for the detection of dopamine. The table summarizes the electrochemical sensing performance of a range of electrodes that have been developed for detecting dopamine. It also presents a brief overview of the key parameters and characteristics of these electrodes, such as their sensitivity, and detection limit. Upon analyzing the information presented in the table, it is observed that the newly developed Cu–Fe NPs/ZEA/Gr thin film electrode exhibits promising results in terms of its electrochemical sensing performance for detecting dopamine. It is important to note that the performance of the Cu–Fe NPs/ZEA/Gr thin film electrode is superior to some of the previously developed electrodes, as suggested by the data presented in the table. These findings highlight the potential of the Cu–Fe NPs/ZEA/Gr thin film electrode as a reliable and effective tool for dopamine detection and suggest that further research and development in this area could lead to significant advances in the field of electrochemical sensing.

**Table 1:** Comparison of the Electrochemical Performance of previously reported dopamine Sensors.

Material	Sensitivity (μA μM^−1^ cm^−2^)	LOD (μM)	Reference
GN/PEDOT/GCE	9 × 10^−3^	54 × 10^−3^	[18]
Fe_2_O_3_–NiO@GO/GCE	1.68 × 10^-−1^	5 × 10^−3^	[19]
Fe_3_O_4_@PPy/rGO	8.35 × 10^-−1^	6.3 × 10^−2^	[20]
PANI/TRGO-700	6.7	4.3 × 10^−1^	[21]
GCE/pyrrolic-N-rGO	3.51	3.35 × 10^−1^	[22]
Cu–Fe NPs/ZEA/Gr	1.97	0.058	This study

#### 3.3.5 Stability and reproducibility of the sensor for detection of dopamine

Long-term storage stability is another requirement that a sensor must satisfy. In 40 mM dopamine, the long-term stability of the produced Cu–Fe NPs/ZEA/Gr thin film has also been examined. When not being used, the modified electrode was stored at room temperature. After two weeks of storage, the electrode still exhibited 98% of its initial reaction as in previous studies mentioned above, and it could sustain 95% of current even after a month. No reaction was measured over a longer period. The reproducibility of the electrode was estimated by measuring responses to 40 mM dopamine solution for 15 successive measurements which exhibits the peak current at ± 0.53 mA which was illustrated in Fig. 12 (C) This demonstrates the long-term durability, stability and reproducibility of the newly developed electrode.

## 4 Conclusion and perspectives

The objective of this research is to gain insights into the rapidly growing global issues and mitigate the risk of disorders. Early detection of diseases is a crucial challenge, and one of them is the neurological disorders, such as anxiety, stress, and depression caused by the faulty production and release of neurotransmitters. Dopamine, a major neurotransmitter that regulates movements and emotions, has a significant impact on people with these conditions. High levels of dopamine can be used as a marker for detecting diseases such as Parkinson's, schizophrenia, anxiety and behavioural disorders. A novel nano-structured electrode, Cu–Fe NPs/ZEA/Gr thin film, was presented for the electrochemical determination of DA. In the initial segment Cu–Fe bimetallic nanoparticles were synthesized effectively. This was blended with ZEA and graphene to produce a novel nanocomposite. The XRD, SEM and other characterization analysis confirms the formation of two-dimensional Cu–Fe NPs/ZEA/Gr nanocomposite thin film. The electrochemical activity of the sensor examined using cyclic voltammetry displays brilliant performances with good sensitivity, stability, reproducibility and limit of detection. Meanwhile, Cu–Fe NPs/ZEA/Gr exhibited higher peak current compared with Cu–Fe NPs and Cu–Fe NPs/ZEA in determination of DA. Additionally, the results showed that the current response of the sensor increased linearly with increasing dopamine concentration and scan rate, demonstrating the feasibility of the Cu–Fe NPs/ZEA/Gr thin film electrode for the electrochemical determination of DA. The high surface area and improved electrical conductivity of the electrode due to the presence of ZEA and graphene played a crucial role in enhancing the electrocatalytic activity. The proposed sensor could provide a simple, rapid and cost-effective alternative for the detection of dopamine and has great potential for application in the diagnosis of neurological disorders and other diseases associated with the imbalance of dopamine levels in the body. Overall, this study highlights the significance of developing novel nano-structured electrodes for the electrochemical determination of neurotransmitters, and the potential for their application in the field of medical diagnosis. The bio-sensor developed in this research can be used to measure dopamine levels in blood and serum and has the potential to be integrated into a handheld device connected to the Internet of Things (IOT) in the future. This device can convert raw data into a digital display, allowing physicians to monitor patients remotely, without the need for human intervention. The system will be beneficial to humanity as it can dynamically communicate and analyze data with other networks.

## Author contribution statement

Navashree Nagarajan: Conceived and designed the experiments; Performed the experiments; Analyzed and interpreted the data; Contributed reagents, materials, analysis tools or data; Wrote the paper.

Parthasarathy Panchatcharam: Contributed reagents, materials, analysis tools or data.

## Data availability statement

Data will be made available on request.

## Additional information

No additional information is available for this paper.

## Declaration of competing interest

The authors declare that they have no known competing financial interests or personal relationships that could have appeared to influence the work reported in this paper.
